# Unveiling the Hidden Links: Anatomical and Radiological Insights into Primary Hip Osteoarthritis

**DOI:** 10.3390/jpm14091004

**Published:** 2024-09-20

**Authors:** Valerio Tiburzi, Carlo Ciccullo, Luca Farinelli, Marco Di Carlo, Fausto Salaffi, Francesca Bandinelli, Antonio Pompilio Gigante

**Affiliations:** 1 Clinical Ortopaedics, Department of Clinical and Molecular Sciences, Politecnica University of Marche, 60126 Ancona, Italyl.farinelli@pm.univpm.it (L.F.); a.p.gigante@staff.univpm.it (A.P.G.); 2 Rheumatology Unit, Department of Clinical and Molecular Sciences, Politecnica University of Marche, Ospedale “Carlo Urbani”—AST Ancona, 60035 Jesi, Italy; marco.dicarlo@staff.univpm.it (M.D.C.); f.salaffi@staff.univpm.it (F.S.); 3 Department, Santa Maria Nuova Hospital, Usl Tuscany Center, 50122 Florence, Italy; francesca.bandinelli@uslcentro.toscana.it; 4 IRCCS INRCA, 60126 Ancona, Italy

**Keywords:** hip osteoarthritis, radiography, spinopelvic alignment, anatomical pathological condition

## Abstract

Background: Hip osteoarthritis (HOA) is a disease with globally rising incidence that leads to disability and morbidity, overall, in older populations, and might be primary or secondary to numerous risk factors. The most common idiopathic HOA is generally a diagnosis of exclusion, with pathogenetic mechanisms largely still misunderstood. We aimed to investigate the correlation between femoral–acetabular and spinopelvic anatomical and computed tomography (CT) characteristics, and the presence of primary OA. Methods: We retrospectively analyzed CT scans from 2019 to 2021, excluding patients under 45 years or with conditions affecting the pelvis, sacrum, or lower limbs. Femoral, acetabular, and spinopelvic parameters were measured; signs of OA were analyzed in the hip and knee joints. Patients were categorized into two groups: A (isolated hip OA) and B (no OA); patients with hip OA, also presenting knee OA, were excluded from this study. Results: In total, 232 cases were examined; statistical analyses compared CT parameters between 129 subjects from Group A and 103 patients of Group B. Group A showed a mean femoral version of 16 ± 4.53 degrees, significantly higher than Group B’s 13.16 ± 4.37 degrees (*p* = 0.0001). Other parameters showed no significant differences. Conclusion: This study highlights an association between femoral version and primary hip OA.

## 1. Introduction

Osteoarthritis (OA) is one of the leading causes of disability and morbidity globally. According to the Prevalence Trends of Site-Specific Osteoarthritis from the Global Burden of Disease Study 2019, the prevalence of OA increased by 113.25% over a decade, rising from 247.51 million cases in 1990 to 527.81 million cases in 2019 [1]. This rising prevalence can be attributed to an aging population and the growing prevalence of metabolic and inflammatory risk factors [1,2,3]. 

OA accounts for a significant number of healthcare visits, knee and hip replacements, and general hospital and rehabilitation costs [4,5]. Additional expenses associated with OA include those not directly related to healthcare, such as loss of work productivity and formal and informal care costs due to the limited independence of affected individuals; in the UK, the total healthcare cost of OA is estimated to exceed GBP 1 billion (2010) [6]. 

The hip is the second most frequently affected joint by OA following the knee. The OA process involves the progressive loss of articular cartilage, subchondral cysts, osteophyte formation, periarticular ligamentous laxity, muscle weakness, and possible synovial inflammation. From a clinical perspective, the condition is characterized by groin pain, joint stiffness, and loss of function [7,8], which collectively serve as the primary indication for hip arthroplasty. The prevalence of hip OA in Caucasian groups is approximately ten times the prevalence of hip OA in Asian populations of the same age and sex [9].

Hip OA can be etiologically categorized into primary and secondary forms. Secondary OA results from known causes that alter the cartilage environment, including trauma, congenital or developmental joint abnormalities, metabolic defects, infections, endocrine diseases, neuropathic conditions, and other disorders that are able to modify the normal structure and function of hyaline cartilage [2,10]. 

Primary or idiopathic OA is generally a diagnosis of exclusion and is believed to account for most hip OA cases. Despite its high prevalence and global impact, the pathogenetic mechanism of this condition remains poorly understood. Several variables, including age, sex, and bone quality, have been identified as potential contributors to the development of this pathology. However, the precise role of these factors remains unclear [11]. 

Biomechanical principles for the development of hip OA are based on force transmission calculations, positing that cartilage degeneration starts from concentric or eccentric overload [11,12]. The traditional standard radiography of these alterations always represents the first approach standard in daily clinical practice. However, more modern instrumental techniques, in particular computed tomography (CT), with 3D reconstruction, may be employed to enhance and complete the diagnosis [13,14,15,16].

Recent studies have supported the hypothesis that primary OA can be associated with developmental anatomical abnormalities, particularly in relation to femoral anteversion, acetabulum anteversion, and femoral–acetabular impingement [17,18,19,20,21]. 

There has also been a correlation between femoral anteversion and the development of hip dysplasia [22].

Therefore, this study aims to investigate the potential correlation between specific anatomical characteristics based on CT scans, like the inclination and version of femur and acetabulum or the spinopelvic alignment, in primary hip OA.

## 2. Materials and Methods

### 2.1. Study Design and Participants

We retrospectively cross-sectionally studied 506 patients from Riuniti Torrette Hospital of Ancona, Politecnica University of Marche, Italy, from 1 January 2019 to 31 December 2021.

For this research, the mandatory inclusion criteria were the availability, for each patient examined, of CT examination that allowed for a clear visualization of the hip and knee joints, as well as the pelvis and sacrum. Therefore, pelvic and lower-limb CT studies and total body CTs performed during this study period, for several reasons, were collected from our unit’s unified database. These causes included suspected oncological pathology, investigation of vascular diseases like thrombi and aneurysms, skeletal examination after trauma, diagnostic investigations for chronic unexplained pain, follow up after medical or surgical treatment, and screening for occult disease in high-risk patients, as well as pre-operative assessments, functional evaluations for sports, or for insurance purposes. Patients under 45 years old at the time of the radiological exam were excluded. Additionally, patients with any internal fixation devices in the spine, pelvis, or lower limbs, or with evident surgical outcomes, osteotomies, or clear skeletal deformities, were excluded from this study. The presence of metastases or neoplasms of any kind in the examined areas was also considered an exclusion criterion. Other exclusion criteria included the presence of joint prostheses in one or both limbs, as well as the results of limb amputations or parts thereof. The CT images were carefully examined individually by three different experienced orthopedics to avoid mistakes in identifying exclusion criteria and to minimize acquisition bias. Privacy and informed consent were obtained from the participants for the collection and publication of anonymous clinical data, according to regional guidelines and Italian Health Ministry legislation (22 April 2021, no. 52), and to the ethical standards of the Declaration of Helsinki of 1964 and its subsequent amendments. This research has been approved by our local Institutional Review Board (Comitato Etico Regionale delle Marche (CERM)—registration number 204/2021, approved on 29 July 2021).

### 2.2. Measurements

The CT scans of subjects considered eligible for this study were carefully analyzed for the subsequent measurement of the femoral, acetabular, and spinopelvic parameters under investigation. 

**Figure 1:**
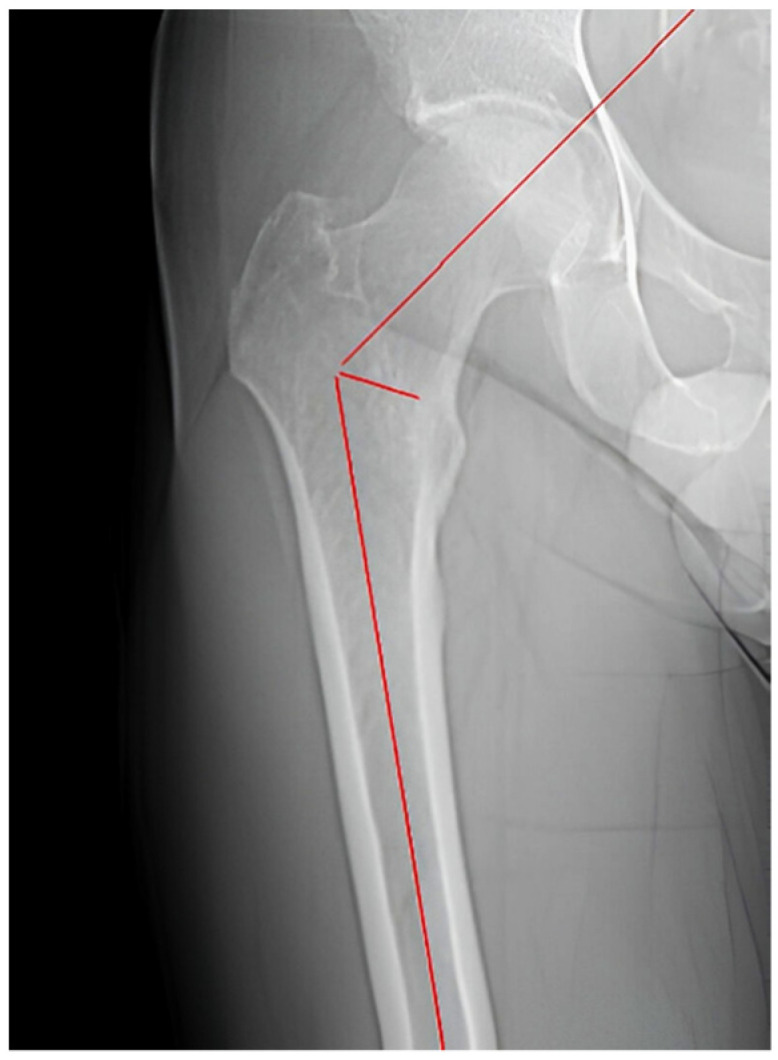
Femoral inclination measurement.

The femoral inclination angle, or femoral neck-shaft angle, is defined as the angle described in the frontal plane between the anatomical axis of the femoral diaphysis and the axis of the femoral neck. It was measured in the CT scan by tracing these lines through the coronal slice showing the greatest transverse width, ensuring they passed through the center of the femoral canal (Figure 1). 

**Figure 2:**
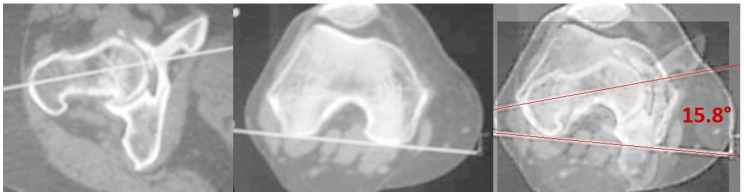
Femoral version measurement. The angle was calculated by overlaying the axial scans of the femoral neck axis and the posterior intercondylar line.

Femoral version, also known as the measurement of femoral torsion, is the angular value defined between the axis of the femoral neck and the posterior intercondylar line on the transverse plane. The measurement was performed by overlaying the axial scan of the distal femur and its tangent line posteriorly to the condyles with the one identifying the axis of the femoral neck (Figure 2). The resulting angle was recorded in the database.

**Figure 3:**
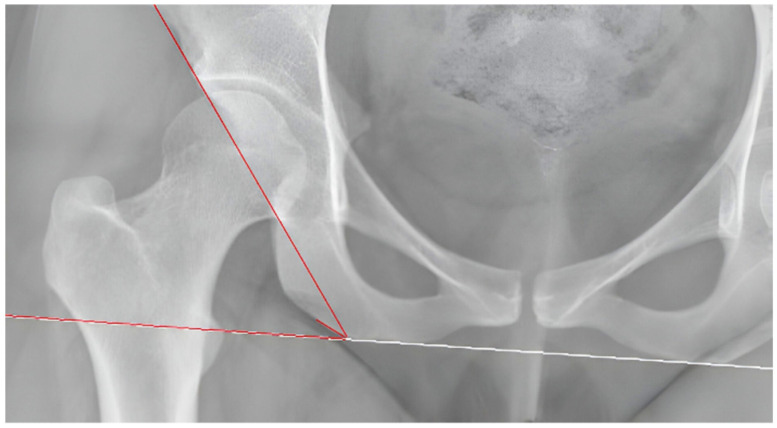
Acetabular inclination measurement. The horizontal reference was set as the tangent line to the ischial tuberosity.

The acetabular inclination angle is defined as the angular measurement between two lines: the tangent line, in the frontal plane, to the upper and lower margins of the acetabulum, and the horizontal line. For the purposes of this study, it was measured on the anterior–posterior (AP) X-ray, which serves as the scout for the CT acquisition. The horizontal reference was set as the tangent line to the ischial tuberosity to normalize the measurement, accounting for any malrotation of the patient during the CT imaging acquisition (Figure 3).

**Figure 4:**
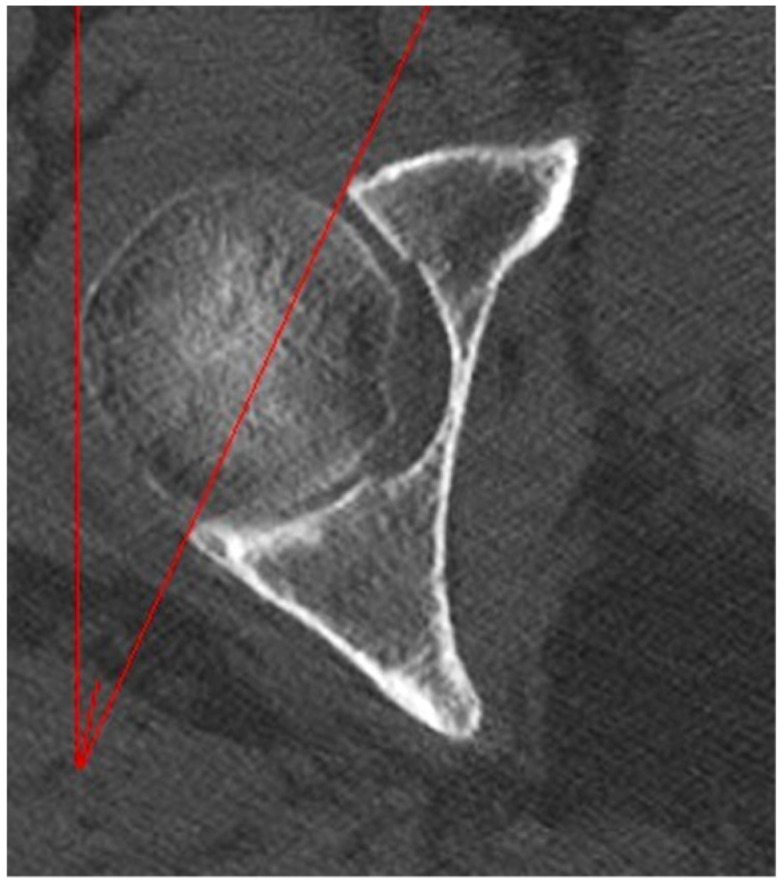
Acetabular version measurement.

The acetabular version is an angular measure that defines the orientation of the acetabular cavity in the axial plane. Its measurement was performed by identifying the axial scan passing through the center of the femoral head and calculating the resulting angle between two lines: the reference antero-posterior axis and the line tangent to the anterior and posterior margins of the acetabulum (Figure 4). 

**Figure 5:**
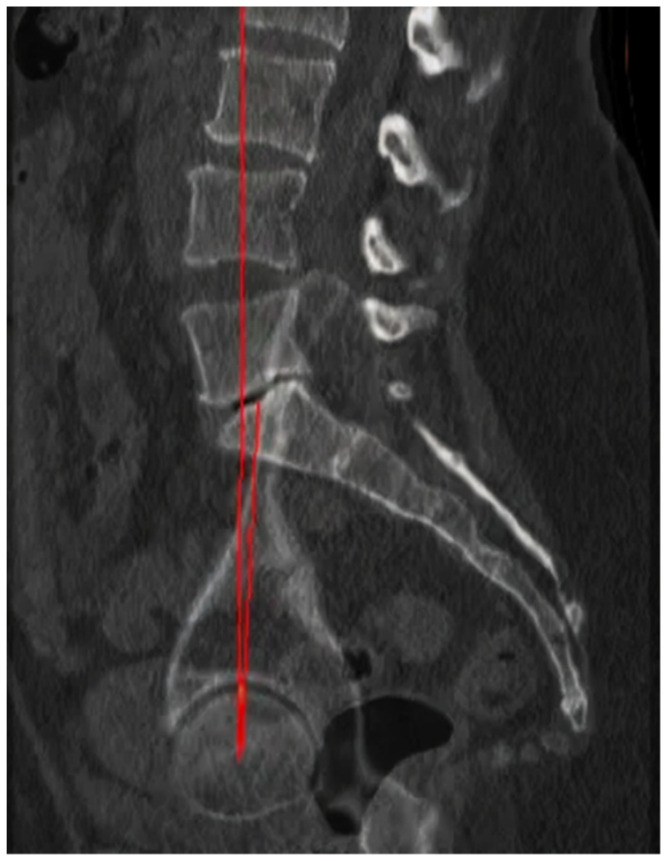
Pelvic tilt measurement. The angle was calculated by overlaying the sagittal scans passing through the center of the sacral promontory and the center of the femoral head.

Pelvic tilt (PT) is an angular parameter created by a line running from the midpoint of the sacral endplate to the center of the bifemoral heads and the vertical axis. The pelvic tilt was calculated by overlaying the sagittal CT scan passing through the center of the sacral promontory and the CT scan passing through the center of the femoral head, then tracing the axis connecting the center of femoral rotation with the midpoint of the sacral roof relative to the vertical line (Figure 5). 

**Figure 6:**
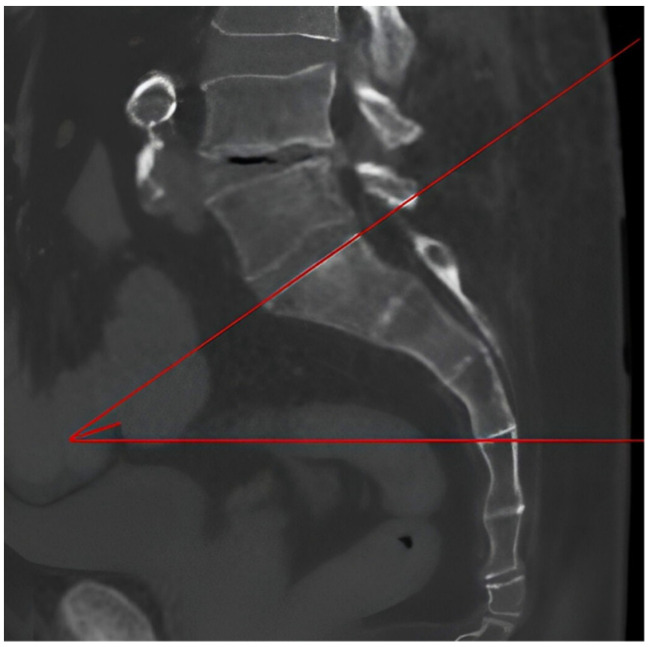
Sacral slope measurement.

The sacral slope (SS) is a radiological parameter that describes the inclination of the sacrum relative to the horizontal plane. It is delineated by the intersection of a line tangent to the sacral plate with the horizon. This was measured on the sagittal scan passing through the center of the sacrum by drawing the tangent line to the upper margin of first sacral vertebra(S1) and calculating its angle relative to the horizontal axis (Figure 6).

**Figure 7:**
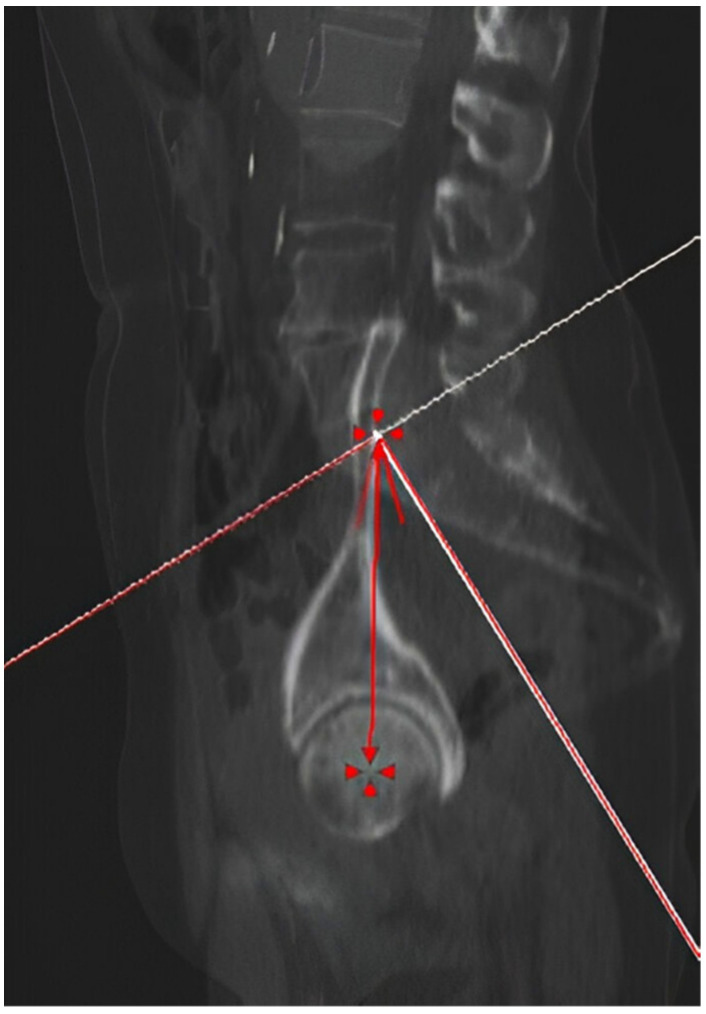
Pelvic incidence measurement. The angle was calculated by overlaying the sagittal scans passing through the center of the sacral promontory and the center of the femoral head. The tangent line to the sacral roof and perpendicular to its midpoint, were drawn (white lines). The resulting angle between the perpendicular line and the center of the femoral head was recorded in the database.

Pelvic incidence (PI) is a radiographic angular value defined by two lines: the first perpendicular to the tangent of the sacral roof at its midpoint, and the second one connecting this point to the hip’s center of rotation. The measurement was performed by overlapping the two sagittal radiographic slices passing through the center of the sacrum and the center of the femoral head and then identifying the described lines and the resulting angle. The value obtained for pelvic incidence was in the end checked by summing PT and SS for further validation (Figure 7).

The CT scans of the hip and knee joints were examined. Signs of OA were identified, distinguishing for each subject enrolled in this study the presence or absence of hip and knee OA. The diagnosis of OA was performed in the same way as patient enrollment: through the independent analysis of radiological images by 3 authors, and the subsequent comparison of data. Discordant assessments were then reassessed collegially. The patients were classified into three groups according to OA diagnosis: Group A, with the presence of hip OA and absence of knee OA; Group B, with the absence of hip OA and absence of knee OA; and Group C, with the presence of hip OA and presence of knee OA. Group A represents the study group, while Group B constitutes the control group. Group C, which includes subjects with OA in both joints, was considered for exclusion from this study.

### 2.3. Statistical Analysis

The calculated sample size of population, based on a similar study [10], achieved >80% statistical power. Descriptive statistics were expressed as means and standard deviations (SDs) for continuous parameters and percentages for categorical variables, as appropriate. The Kolmogorov–Smirnov and Shapiro–Wilk tests were used to evaluate the distribution of the single cohorts’ variables. The data of the different groups examined, with normal distribution, were assessed using unpaired *t*-tests, as appropriate. Categorical data were compared among groups using the Chi-square test, as appropriate. The level of statistical significance was set at *p* value < 0.05. All statistical analyses were performed using GraphPad prism 8.0. The data reported in this study conform to STROBE guidelines [23].

## 3. Results

**Figure 8:**
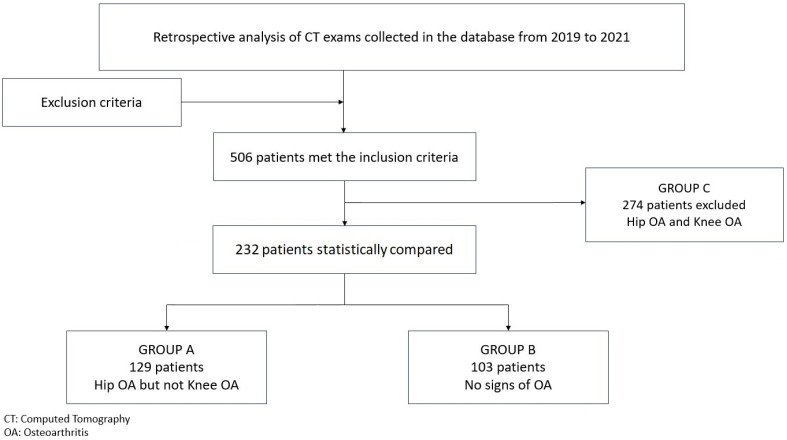
Study flow chart. Patients were classified into three groups according to OA diagnosis: Group A, presence of hip OA, absence of knee OA; Group B, absence of hip OA, absence of knee OA; Group C, presence of hip OA, presence of knee OA. Group A represents the study group, while Group B constitutes the control group. Group C, which includes subjects with OA in both joints, was considered for exclusion from this study.

During this study period, 506 patients met the inclusion criteria. Based on the independent assessment of CT studies, the patients were divided into three groups as follows: 129 patients in Group A, 103 patients in Group B, and 274 patients in Group C. Group C was consequently excluded according to the inclusion criteria established in the study design. The remaining 232 patients were compared for the described variables. A flow chart of this study is shown in Figure 8.

### 3.1. Differences in Femoral Parameters between Group A (Hip OA) and Group B (Absence of Hip OA)

**Figure 9:**
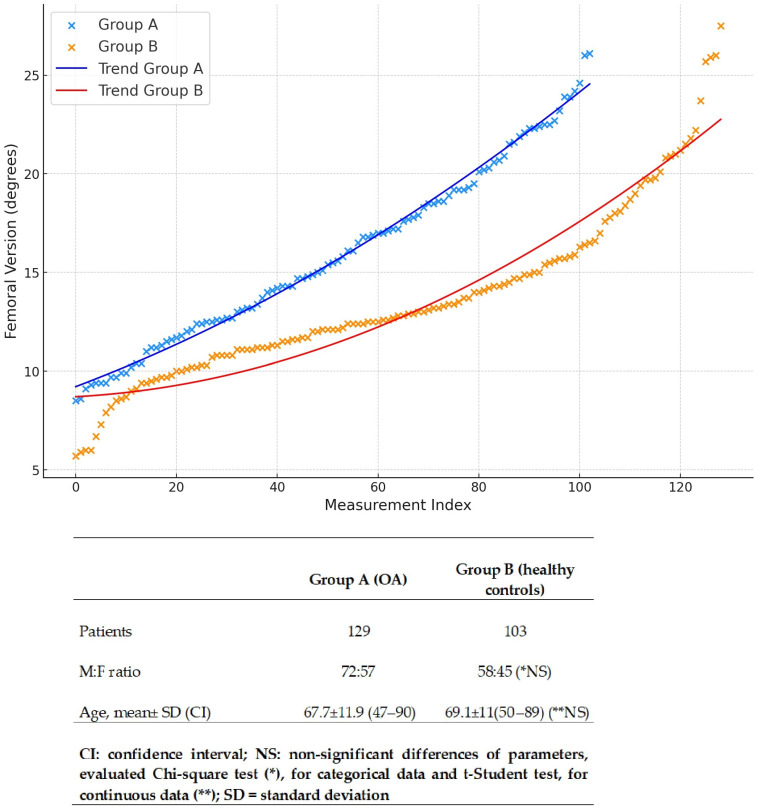
Ordered distribution of femoral version in Group A (hip OA) and Group B (controls) expressed in means, SDs (standard deviations), and CIs (confidence intervals) with a non-significant distribution of sex and age.

Group A consisted of 57 women and 72 men, aged between 47 and 90 years old, with a mean age of 67.7 ± 11.9 years. Group B consisted of 45 women and 58 men, aged between 50 and 89 years old, with a mean age of 69.1 ± 11 years, with no statistical difference in parameters (Figure 9).

**Figure 10:**
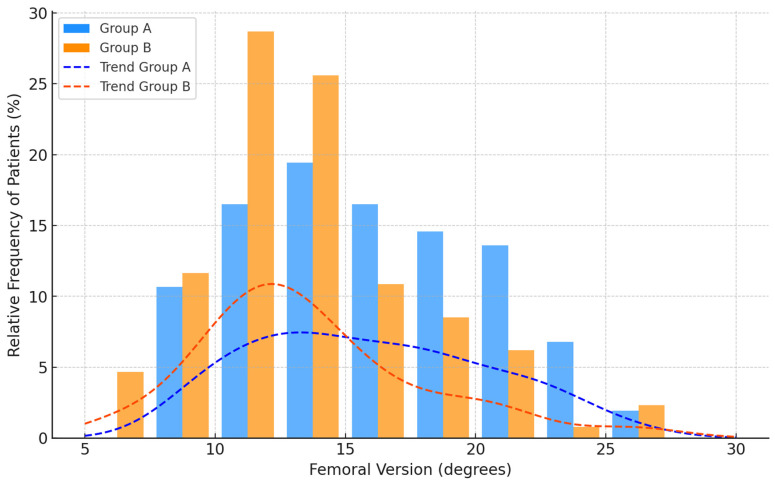
The distribution of patients in Group A (hip OA) and Group B (controls) relative to femoral version values, expressed as a percentage of the number of patients per group.

Group A displayed femoral parameters with a mean femoral version of 16.00 ± 4.53 degrees and a mean femoral inclination of 128.46 ± 7.16 degrees. In Group B, the mean femoral version was 13.16 ± 4.37 degrees, and the mean femoral inclination was 129.95 ± 6.86 degrees (Figure 9 and Figure 10). The statistical comparison of these values, performed using the unpaired Student’s *t*-test, yielded *p*-values of 0.0001 and 0.107, respectively (Table 1).

### 3.2. Differences in Acetabular Parameters between Group A and Group B

For the acetabular parameters, the mean version in the first group was 19.60 ± 4.19 degrees, whereas in the second cohort, it was 20.37 ± 4.56 degrees (*p*-value = 0.180). The mean acetabular inclination was 57.15 ± 4.68 degrees in Group A and 56.39 ± 4.57 degrees in Group B (*p*-value = 0.216) (Table 1)

### 3.3. Differences in Spino-Pelvic Parameters between Group A and Group B

The calculated spinopelvic parameters showed a mean sacral slope of 41.62 ± 7.7 degrees in Group A and 40.35 ± 6.2 degrees in Group B (*p*-value = 0.164), while the mean pelvic tilt was 9.84 ± 4.68 degrees in the first group compared to 9.91 ± 5.45 degrees in the second one (*p*-value = 0.916). Lastly, the pelvic incidence was on average 50.57 ± 10.24 degrees for Group A and 50.11 ± 9.8 degrees for Group B, with a resulting *p*-value of 0.728. (Table 1)

## 4. Discussion

Primary idiopathic OA constitutes the prevailing form of hip OA, with a major epidemiological impact and significant clinical, social, and economic consequences. Despite the scale of the problem, many aspects of its pathogenetic mechanisms remain unclear. Improving our understanding of its etiology and potential risk factors could lead to significant therapeutic and preventive advancements.

This study highlights a possible correlation between idiopathic hip OA and femoral version, exploring anatomical factors as a potential etiological contributor to this disease. Inizio moduloFine modulo

Based on the study results, the only variable that showed a statistically significant value in the comparison between the two groups was femoral version. Specifically, Group A, the group consisting of patients with hip OA, presented a higher mean femoral version value compared to Group B (16 ± 4.53 vs. 13.16 ± 4.37), the control group, characterized by patients without radiological evidence of OA in the hip and knee joints. This result suggests that femoral version may be a relevant parameter in the association with idiopathic primary hip OA.

A systematic review and meta-analysis by Parker et al. [17] demonstrated results consistent with those of this study; specifically, an analysis of 19 studies indicated that patients with a femoral version greater than 14 degrees are more likely to develop hip OA, with significantly higher values (14°–24°) corresponding to higher grades of OA, according to the Kellgren–Lawrence classification. Similarly, a pilot study by Terjesen et al. [24] found that individuals diagnosed with hip OA exhibited higher average femoral version values compared to the control group (17.5° vs. 13°).

Conversely, the study by Alexander et al. [25] showed that in adolescents, increased femoral anteversion, and the resultant gait alteration with in-toeing, do not necessarily lead to altered forces on the hip joint, and thus predisposition to early wear. Compensatory mechanisms may be involved, suggesting that kinetics, rather than anatomical features, should be considered.

None of the other parameters analyzed, related to the femur or acetabulum, appear to provide a significant difference based on the established inclusion and exclusion criteria. This is equally important when considering pathological conditions predisposing people to OA, such as congenital hip dysplasia. In this pathological condition, various anatomical alterations of the hip joint coexist, such as hypoplasia, the flattening and lateralization of the femoral head, increased femoral anteversion, and increased acetabular index, resulting in a loss of articular congruence. Based on these data, however, it is plausible that femoral anteversion itself is the parameter most responsible for joint degeneration, a concept also emphasized in the study published by Li et al. [26].

A recent article published by Rezaei et al. [18] demonstrates that patients with a lower relative acetabular version (<15 degrees) experience symptomatic OA approximately 4 years earlier than the control group. However, this finding emphasizes the clinical aspect and the pain that leads patients to require hip replacement surgery, and does not necessarily conflict with the purely radiographic data of this study.

Neither of the spinopelvic parameters showed a significant difference in the comparison between the two groups under study. Although they do not appear to be predisposing factors for the development of osteoarthritis, they can influence its treatment. It is increasingly recognized that they should be considered in the surgical planning of prosthetic replacement due to their impact on postoperative outcomes. Specifically, it has been highlighted that individuals with alterations in spinopelvic parameters associated with flatback deformity and spinal stiffness are at a higher risk of dislocation after total hip arthroplasty [27,28].

Each parameter examined in this study has been recognized in the scientific literature as having a direct or indirect pathogenetic role in the hip joint, thus justifying its inclusion among the variables analyzed in this research.

Femoral version represents the degree of femoral torsion along its axis, with normal values ranging between 8 and 15 degrees. The clinical implications of altered femoral version are profound [29]. Increased anteversion has been associated with anterior cruciate ligament (ACL) injuries due to altered knee biomechanics [30]. Conversely, a reduction in anteversion or retroversion can exacerbate conditions like femoro-acetabular impingement (FAI) by altering hip joint congruence and increasing impingement risk [31]. In pediatric populations, rotational abnormalities such as excessive anteversion often resolve spontaneously with growth. The precise measurement of this parameter requires radiographic examinations; however, they can be evaluated clinically through the Craig/Ryder test and gait analysis. Excessive anteversion is associated with in-toeing gait, while retroversion, which is less prevalent, presents without toeing. In rare instances of persistent severe cases surgical treatment in the form of derotation osteotomy may be required [32,33].

The femoral inclination, or femoral neck-shaft angle (NSA), is defined as the angle between the axis of the femoral neck and the axis of the femoral shaft. Normal values range between 120 and 135 degrees [34]. Both increased NSA (coxa valga) and decreased NSA (coxa vara) can result from congenital conditions, malformation syndromes, or acquired post-traumatic forms. Most cases are clinically asymptomatic, but significant variations in the inclination angle can have important implications. Coxa vara typically presents with a shortened limb, limited abduction, and a Trendelenburg gait due to weakened abductor muscles. Biomechanically, this results in increased shear forces across the hip joint, potentially predisposing patients to early osteoarthritis [35]. Coxa valga increases compressive forces on the lateral aspect of the acetabulum while reducing the effectiveness of medial support structures. This malalignment can lead to secondary complications, such as labral tears or early-onset osteoarthritis, due to altered load distribution across the hip joint.

Acetabular version is an anatomical parameter that describes the orientation of the acetabulum in the axial plane. It is measured as the angle between a line tangent to the anterior and posterior margins of the acetabulum and the sagittal axis of the body. In normal conditions, acetabular version ranges between 15° and 20° (physiological anteversion). Excessive acetabular anteversion or acetabular retroversion can reduce joint containment and predispose patients to dislocations, or cause femoroacetabular impingement (FAI), resulting in reduced range of motion and pain [36].

The spinopelvic parameters demonstrated a direct correlation with the pelvis, postural alignment, and gait, starting from the profound changes throughout human evolution. The adoption of an upright posture by humans resulted in a widening and verticalization of the pelvis, along with the development of characteristic spinal curves in the sagittal plane [35]. Therefore, these parameters were also analyzed in the investigation of potential implications in the development of primary hip osteoarthritis.

Pelvic incidence (PI) is an anatomical parameter that remains constant after skeletal maturity. It is measured as the angle between a line perpendicular to the midpoint of the sacral endplate and a line connecting this point to the axis of the femoral heads. In conditions like lumbar spondylolisthesis, higher PI values are associated with more severe slippage. PI influences pelvic tilt (PT) and sacral slope (SS) through the equation PI = PT + SS. Pelvic tilt and sacral slope are dynamic parameters that adjust to maintain sagittal balance. Sacral slope decreases as PT increases, and vice versa, maintaining the relationship defined by PI. Sacral slope is the angle between the horizontal plane and the sacral endplate. A higher SS indicates a more anteriorly tilted sacrum, correlating with increased lumbar lordosis. Pelvic tilt is the angle between the vertical plane and a line connecting the midpoint of the sacral endplate to the axis of the femoral heads. Increased PT reflects posterior pelvic rotation, often seen as a compensatory mechanism in spinal pathologies like kyphosis or spondylolisthesis [37].

Although both pelvic tilt (PT) and sacral slope (SS) are dynamic parameters whose values depend on the patient’s position in space, all radiographic measurements were carried out in the supine position, as required by the CT gantry. This decision was mainly driven by ethical considerations, particularly the requirement, in a prospective study involving scans in different postures, to conduct CT exams of the pelvis and lower extremities to potentially healthy individuals leading to considerable ionizing radiation exposure. This consideration also mandated the use of only 2D methods or multiplanar CT scans instead of computerized measurements on potentially more accurate 3D models [38].

In defining the exclusion criteria for this study, a lower age limit of 45 years was set for the patients examined. This was to avoid excluding patients who, although predisposed to developing idiopathic primary osteoarthritis, did not yet have radiologically evident signs of pathology such as joint space narrowing, subchondral bone sclerosis, or the presence of osteophytes and/or geodes, and could thus be falsely negative in the evaluation. Patients with bone alterations, skeletal deformities, or neoplasms, that were identifiable directly or indirectly though imaging, were excluded, as they could potentially be attributed to secondary forms of osteoarthritis.

In structuring the study protocol, it was decided to exclude patients exhibiting clear signs of osteoarthritis in both examined regions, the hip and knee, to minimize confounding factors (Group C). Patients with multi-joint osteoarthritis might reflect genetic predispositions to accelerated joint degeneration or secondary forms of osteoarthritis. Since all subjects with knee osteoarthritis were excluded, variables directly related to this joint, such as patellar alignment, tibial torsion, and similar parameters, were not considered.

These criteria were designed to limit the selection of patients to those with potentially idiopathic hip osteoarthritis.

Patient recruitment for this study focused almost exclusively on radiological factors, limiting the evaluation of other key aspects critical to understanding the patient’s overall medical profile, such as medical history or “social” factors. Numerous factors are known to influence the development and progression of osteoarthritis. For instance, a high BMI is a well-documented risk factor due to its direct mechanical overload on the joint [12]. Overuse linked to occupational or sports activities can influence the progression of osteoarthritis in a specific anatomical region [11]. A patient’s recent medical history might indicate chronic corticosteroid use, the presence of chronic inflammatory conditions affecting the bone and the joint [3,39]. This aspect represents a significant limitation of this study. Moreover, the retrospective nature of patient enrollment and the cross-sectional design represent additional weaknesses of this study. However, we attempted to mitigate this bias by evaluating a large population of patients, screening over 500 subjects, thereby reducing the potential implications of non-assessable factors in this context (pharmacological therapy, BMI, medical history) to a minimal percentage in the study population.

## 5. Conclusions

This study highlights an association between femoral version and primary hip OA, emphasizing the importance of considering femoral version in the assessment and management of patients at risk for or suffering from hip OA.

While this condition is certainly multifactorial in etiology and still largely misunderstood, this finding could represent a small step forward in clarifying its intrinsic mechanisms of development.

Confirming the association between an increased femoral version angle and a higher probability of developing idiopathic hip OA could position this variable among the recognized risk factors for the disease, becoming a parameter for evaluating patients at risk or suggesting their closer monitoring over time. It will also be crucial to examine how this parameter interacts with other known risk factors (age, BMI, physical activity) and how it influences the long-term progression of OA. This could lead to the development of a risk stratification model for the general population, potentially enabling the planning of effective management strategies with a focus on preventive interventions for at-risk patients, such as lifestyle modifications, targeted physiotherapy, or specific infiltrative therapies.

However, further and more extensive studies are required to obtain more concrete data, reassessing this study prospectively on more selectively grouped patients, and including the evaluation of clinical aspects and the severity staging of osteoarthritis.

## Figures and Tables

**Table 1:** Computed tomography measurements and differences between groups.

CT Parameters	Group A (Hip OA)	Group B (Controls)	*p* Value
Femoral Version	16.00° ± 4.53° (15.22°–16.78°)	13.16° ± 4.37° (12.31°–14.01°)	**0.0001**
Femoral Inclination	128.46° ± 7.16° (127.22°–129.70°)	129.95° ± 6.86° (128.61°–131.29°)	0.107
Acetabular Version	19.60° ± 4.19° (18.81°–20.39°)	20.37° ± 4.56° (19.47°–21.27°)	0.180
Acetabular Inclination	57.15° ± 4.68° (56.25°–58.05°)	56.39° ± 4.57° (55.49°–57.29°)	0.215
Sacral Slope	41.62° ± 7.70° (40.27°–42.97°)	40.35° ± 6.20° (39.14°–41.56°)	0.915
Pelvic Tilt	9.80° ± 4.68° (8.90°–10.70°)	9.91° ± 5.45° (8.84°–10.98°)	0.917
Pelvic Incidence	50.57° ± 10.24° (48.77°–52.37°)	50.11° ± 9.80° (48.19°–52.03°)	0.728

Values were expressed in means, SDs (standard deviations), and CIs (confidence intervals); significant differences are in bold; CT: computerized tomography; OA: osteoarthritis.

## Data Availability

The data are available if requested.
